# The neuromuscular and multisystem features of *RYR1*-related malignant hyperthermia and rhabdomyolysis: A study protocol

**DOI:** 10.1097/MD.0000000000026999

**Published:** 2021-08-20

**Authors:** Luuk R. van den Bersselaar, Nick Kruijt, Gert-Jan Scheffer, Lucas van Eijk, Ignacio Malagon, Stan Buckens, José AE Custers, Leonie Helder, Anna Greco, Leo AB Joosten, Baziel GM van Engelen, Nens van Alfen, Sheila Riazi, Susan Treves, Heinz Jungbluth, Marc MJ Snoeck, Nicol C. Voermans

**Affiliations:** a Malignant Hyperthermia Investigation Unit, Department of Anesthesiology, Canisius Wilhelmina Hospital, Nijmegen, The Netherlands; b Department of Neurology, Donders Institute for Brain, Cognition and Behavior, Radboudumc, Nijmegen, The Netherlands; c Department of Anesthesiology, Pain and Palliative Medicine, Radboudumc, Nijmegen, The Netherlands; d Department of Radiology, Radboudumc, Nijmegen, The Netherlands; e Department of Medical Psychology, Radboud Institute for Health Sciences, Radboudumc, Nijmegen, The Netherlands; f Department of Internal Medicine, Radboud Institute of Molecular Life Sciences, Radboudumc, Nijmegen, The Netherlands; g Department of Medical Genetics, Iuliu Haţieganu University of Medicine and Pharmacy, Cluj-Napoca, Romania; h Department of Anesthesiology and Pain Medicine, Malignant Hyperthermia Investigation Unit, University Health Network, University of Toronto, Toronto, Canada; i Department of Biomedicine, University Hospital Basel, Basel, Switzerland; j Department of Paediatric Neurology, Neuromuscular Service, Evelina Children's Hospital, Guy's and St Thomas’ Hospital NHS Foundation Trust; k Randall Centre for Cell and Molecular Biophysics, Muscle Signalling Section, King's College, London, United Kingdom.

**Keywords:** calcium signaling, malignant hyperthermia, myopathy, neuromuscular disorder, rhabdomyolysis, Ryanodine receptor-1, *RYR1*, skeletal muscle cell

## Abstract

**Introduction:**

Malignant hyperthermia (MH) and exertional rhabdomyolysis (ERM) have long been considered episodic phenotypes occurring in response to external triggers in otherwise healthy individuals with variants in *RYR1*. However, recent studies have demonstrated a clinical and histopathological continuum between patients with *RYR1*-related congenital myopathies and those with ERM or MH susceptibility. Furthermore, animal studies have shown non-neuromuscular features such as a mild bleeding disorder and an immunological gain-of-function associated with MH/ERM related *RYR1* variants raising important questions for further research. Awareness of the neuromuscular disease spectrum and potential multisystem involvement in *RYR1*-related MH and ERM is essential to optimize the diagnostic work-up, improve counselling and and future treatment strategies for patients affected by these conditions. This study will examine in detail the nature and severity of continuous disease manifestations and their effect on daily life in patients with *RYR1*-related MH and ERM.

**Methods:**

The study protocol consists of four parts; an online questionnaire study, a clinical observational study, muscle imaging, and specific immunological studies. Patients with *RYR1*-related MH susceptibility and ERM will be included. The imaging, immunological and clinical studies will have a cross-sectional design, while the questionnaire study will be performed three times during a year to assess disease impact, daily living activities, fatigue and pain. The imaging study consists of muscle ultrasound and whole-body magnetic resonance imaging studies. For the immunological studies, peripheral mononuclear blood cells will be isolated for in vitro stimulation with toll-like receptor ligands, to examine the role of the immune system in the pathophysiology of *RYR1*-related MH and ERM.

**Discussion:**

This study will increase knowledge of the full spectrum of neuromuscular and multisystem features of *RYR1*-related MH and ERM and will establish a well-characterized baseline cohort for future studies on *RYR1*-related disorders. The results of this study are expected to improve recognition of *RYR1*-related symptoms, counselling and a more personalized approach to patients affected by these conditions. Furthermore, results will create new insights in the role of the immune system in the pathophysiology of MH and ERM.

**Trial registration:**

This study was pre-registered at ClinicalTrials.gov (ID: NCT04610619).

## 1 Introduction

Variants in *RYR1,* the gene encoding for the ryanodine receptor-1 (RyR1), may lead to disturbances of calcium homeostasis in skeletal muscle and give rise to a wide variety of congenital myopathies including central core disease, multi-minicore disease, centronuclear myopathy and congenital fibre type disproportion.^[1,2]^ Moreover, *RYR1* variants account for a substantial proportion of patients presenting with episodic conditions as exertional rhabdomyolysis (ERM) and malignant hyperthermia (MH), a disorder that clinically manifests as a hypermetabolic crisis after exposure to volatile anesthetics and/or succinylcholine.^[3,4]^ These episodic phenotypes and *RYR1*-related myopathies have long been considered separate entities, however, recent studies have demonstrated a substantial clinical and histopathological overlap in patients with congenital myopathies and ERM or MH susceptibility.^[2,5–7]^

Although many studies on *RYR1-*related phenotypes have focused on the neuromuscular features, recent studies suggest multisystem involvement in *RYR1*-related diseases. A study in a knock-in mouse model with the MH-related *RYR1* variant Y522S showed that this variant leads to prolonged bleeding by altering vascular smooth muscle cell function.^[8]^ Another study in the same mouse model showed that this gain-of-function *RYR1* variant may offer selective immune advantages due to a more efficient specific immune response.^[9]^ A role of *RYR1* in the immune system is also supported by the identification of functional RyR1 s in human B-cells and dendritic cells.^[10–12]^ Furthermore, Epstein-Barr virus-immortalized B-cells from malignant hyperthermia susceptible (MHS) individuals carrying the V2168 M *RYR1* variant are more sensitive to the RyR1 to the RyR1 activator 4-Chloro-m-Cresol (4-CmC), and produced more interleukin (IL)-1β and IL-6 after treatment with 4-CmC and caffeine.^[13]^ This finding provides a potential explanation for the observation that ERM events often occur during or shortly after infections.^[14]^ Overall, these findings suggest multisystem disease manifestations associated with RyR1 dysfunction and raise important questions for further research. Therefore, a detailed study is needed to improve the knowledge of the neuromuscular and multisystem involvement of *RYR1*-related diseases, in patients with MH and ERM. This will enable health care professionals to recognize the whole spectrum of *RYR1*-related disease, thus improving the diagnostic work-up and counselling of patients with *RYR1* variants. Furthermore, better understanding of potentially associated immunological alterations in patients with *RYR1* variants will provide more insights in the immune system as a modifying factor in the pathophysiology of ERM and MH.

## 2 Methods

### 2.1 Study design

This study is a prospective, cross-sectional cohort study, and will be performed from August 2020 to August 2023 at the Radboud University Medical Centre and the Canisius Wilhelmina Hospital, Nijmegen, The Netherlands. The study protocol was approved by the regional medical ethics committee (CMO Arnhem–Nijmegen, registration number 2020-6251) and is pre-registered at ClinicalTrials.gov (ID: NCT04610619).

### 2.2 Objective

The primary objective of this study is:

1. To investigate the full spectrum of disease manifestations of *RYR1*-related MH/ERM to optimize recognition, acknowledgement and counselling of *RYR1*-related complaints.

The secondary objectives are:

1. To establish a well-characterized baseline cohort for future studies on *RYR1*-related disorders;
2. To investigate the role of the immune system as a potentially modifying factor in ERM and MH.

### 2.3 Participants

Subjects will be recruited by the treating physicians from the MH and ERM cohorts at the national MH Investigation Unit in the Canisius Wilhelmina Hospital, Nijmegen and the neuromuscular outpatient clinic of the Radboud University Medical Centre, Nijmegen.

#### 2.3.1 Inclusion criteria

1. A history of MH susceptibility or ERM. MH susceptibility is defined as a pathogenic *RYR1* variant (www.emhg.org/diagnostic-mutations) or a positive in vitro contracture test (IVCT) according to the European Malignant Hyperthermia Group diagnostic guideline.^[15]^ A history of ERM is defined as at least one episode of clinical symptoms of rhabdomyolysis (cramps, myalgia, myoglobinuria, muscle weakness and/or muscle swelling) with a sudden increase (> 10.000 U/L) and a subsequent fall of creatine kinase (CK).^[16]^
2. Genetically confirmed *RYR1* variant classified as pathogenic, likely pathogenic or a variant of unknown significance according to the variant curation expert panel recommendations for *RYR1* pathogenicity classifications in MH susceptibility.^[17]^
3. Speak, read, write and understand Dutch fluently;
4. Age ≥ 18 years old.

#### 2.3.2 Exclusion criteria

Patients with the following characteristics were excluded from participation in this study:

1. Patients with an initial presentation of a *RYR1*-related congenital myopathy, manifesting with muscle weakness;
2. Other (neuromuscular) diseases resulting in muscle weakness;
3. Patients with a contra-indication for magnetic resonance imaging (MRI);
4. Symptoms of angina pectoris;
5. Current malignancy;
6. Patient using systemic steroids for a period > 14 days during the last 3 months;
7. Pregnancy or breast-feeding.

#### 2.3.3 Additional exclusion criteria for the immunological studies

1. Diabetes mellitus;
2. Established diagnosis of immune deficiency;
3. Fever, surgery or trauma in the last 3 months;
4. The use of statins in the last 12 months;
5. Patient using systemic steroids for a period over 14 days during the last 12 months.

### 2.4 Study procedures

**Figure 1:**
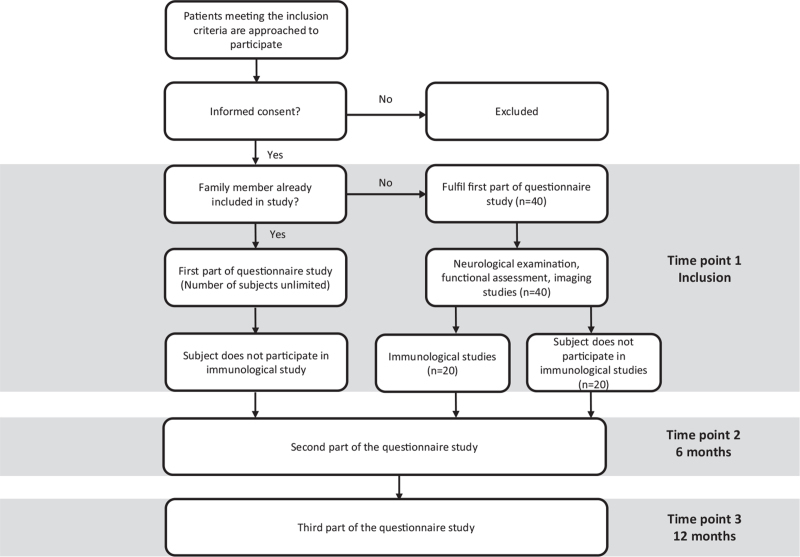
A summary of the study design.

This study consists of four main parts, a questionnaire, clinical, imaging, and immunological part. A total of 20 patients will participate in all study parts while an additional 20 subjects will only participate in the questionnaire, clinical and imaging studies. Furthermore, the questionnaire study is open to all patients meeting the inclusion criteria, without participation in the other study parts. The study design is summarized in Figure 1.

#### 2.4.1 Part 1: Questionnaire studies

Validated questionnaires will be used to asses disease impact including physical activity (International Physical Activity Questionnaire (IPAQ)),^[18,19]^ functional impairment (Sickness Impact Profile (SIP)),^[20,21]^ pain (McGill's Pain Questionnaire (MPQ)),^[22,23]^ perceived fatigue (Checklist Individual Strength (CIS)),^[24,25]^ quality of life (RAND-36),^[26,27]^ mental health (Hospital Anxiety and Depression Scale (HADS)),^[28,29]^ lower urinary tract symptoms (International Prostate Symptom Score (IPSS)),^[30,31]^ increased bleeding tendency (Molecular and Clinical Markers for the Diagnosis and Management of Type 1 von Willebrand disease (MCMDM-1VWD Bleeding Questionnaire)),^[32]^ and worries about the occurrence of a MH or ERM episode (modified version of the Cancer Worry Scale (CWS)).^[33]^ Furthermore, purpose-designed questionnaires focusing on neuromuscular, cardiac and respiratory symptoms will be used as tools to explore topics of interest for future research.

Subjects included in this part of the study will fill out digital questionnaires at three-time points; upon recruitment (t = 0), at 6 months (t = 1) and at 12 months (t = 2). A detailed timeline of the study design of the questionnaire study is given in Table 1.

**Table 1:** Summary of the study design of the questionnaire study.

Questionnaire	Time point 1 (inclusion)	Time point 2 (6 months)	Time point 3 (12 months)
IPAQ ^[18,19]^	x	X	x
SIP ^[20,21]^	x	X	x
MPQ ^[22,23]^	x	X	x
CIS ^[24,25]^	x	X	x
RAND-36 ^[26,27]^	x	X	x
HADS ^[28,29]^	x	x	x
IPSS ^[30,31]^	x		
MCMDM-1VWD Bleeding Questionnaire ^[32]^	x		
Modified CWS ^[33]^	x	x	x
Neuromuscular symptoms questionnaire	x		
Cardiovascular symptoms questionnaire		x	
Respiratory symptoms questionnaire			x

IPAQ = International Physical Activity Questionnaire, SIP = Sickness Impact Profile, MPQ = McGill's Pain Questionnaire, CIS = Checklist Individual Strength, HADS = Hospital Anxiety and Depression Scale IPSS = International Prostate Symptom Score, MCMDM-1 VWD = Molecular and Clinical Markers for the Diagnosis and Management of Type 1 von Willebrand disease, CWS = Cancer Worry Scale.

#### 2.4.2 Part 2: Clinical studies

General information on the medical history, current medication, body weight and height and results from previous investigations (e.g. muscle biopsies, electromyography) will be collected from the electronic health records.

The degree and extent of muscle weakness will be measured using Medical Research Council (MRC)-scores and quantitative myometry using a fixed myometry testing. Hypermobility will be rated using the Bulbena^[34]^ and Beighton score.^[35]^ Respiratory muscle weakness will be tested using spirometry in sitting and supine position (vital capacity, forced vital capacity, forced expiratory volume in the first second). In addition, a timed up and go test,^[36]^ 30 seconds sit to stand test^[37]^ and a six-minute walking test will be performed.^[38]^ Furthermore, resting CK levels will be measured during the visit at the outpatient clinic.

#### 2.4.3 Part 3: Imaging studies

The imaging study consists of two parts, a muscle ultrasound and a muscle MRI. Previous studies in facioscapulohumeral dystrophy have shown that these techniques are complementary, since ultrasound is most sensitive in early stage of the disease while MRI sensitivity increases in later disease stages.^[39]^

##### 2.4.3.1 Ultrasound

A quantitative and qualitative muscle ultrasound examination will be performed using an Esaote MylabTwice ultrasound scanner (Esaote SpA, Genoa, Italy) with a 3–13 MHz broadband linear transducer. The protocol will include a bilateral ultrasound of the musculus trapezius, deltoideus, biceps brachii, thoracic and lumbar erector spinae, iliopsoas, proximal vastus lateralis, biceps femoris and gastrocnemius (medial head). The ultrasound protocol was previously reported elsewhere.^[40]^ Briefly, a strict preset for system settings will be used to ensure reproducibility. Three consecutive measurements will be performed, and results will be averaged offline. For every muscle, a region of interest is drawn manually to calculate the mean echogenicity using the histogram function of a custom image analysis package. Using age, sex and weight corrected reference values, Z-scores (the number of standard deviations from the mean) will be calculated for each muscle. Z-scores below 2 (i.e. below the population 95th percentile) are considered normal.

A semi-quantitative assessment of the images will be performed using the Heckmatt rating scale which ranges from one (normal echo-intensity) to four (severely increased echo-intensity with absent bone reflection) by an experienced neuromuscular ultra-sonographer.^[41]^

##### 2.4.3.2 MRI

A whole-body MRI will be performed using a 1,5 Tesla MR system (Avanto Fit, Siemens, Erlangen, Germany). Volumetric Interpolated Breath-hold Examination (VIBE) 2-point DIXON (proton density weighted) and Short-TI Inversion Recovery (STIR) sequences will be acquired. STIR images will be used as an indicator for edema and/or inflammation. The VIBE 2-point DIXON images will be used to evaluate muscle volume and presence of fatty infiltration.

#### 2.4.4 Part 4: Immunology

Immunological investigations will include a set of laboratory studies using blood samples from a subset of 20 *RYR1* patients and 40 age and sex matched healthy controls. Healthy controls will be recruited amongst volunteers from the staff members of the Radboud University Medical Centre and the Canisius Wilhelmina Hospital.

Peripheral mononuclear blood cells (PBMC) will be isolated from EDTA blood of *RYR1* patients and healthy controls. PBMCs will be stimulated for 24 hours with the Toll-like receptor (TLR) ligands *Escherichia coli* lipopolysaccharide (LPS) and heat-killed Candida in the presence of RyR1 agonists 4-CmC and caffeine and/or the RyR1 antagonist dantrolene. Cytokine levels (IL-1β, IL-6 and TNF-α) in the supernatant of the ERM/MHS subjects will be compared to healthy controls.^[9,12,13]^ Additionally, circulating cytokines (IL-1β, IL-6) will be measured in all participating patients.^[9]^

### 2.5 Statistical analysis

Continuous variables will be summarized by their mean and standard deviation or median and interquartile ranges depending on its distribution. Categorical data, including ordinal data, will be summarized in terms of percentages. Results from the questionnaire studies and cytokine production from *RYR1* patients will be compared to normal values and (if applicable) healthy controls using the students *t*-test.

Due to the explorative nature of this study we did not perform a power calculation. For the clinical and imaging studies, 40 subjects will be included since previous studies and case series with similar sample size showed a substantial clinical and histopathological overlap in patients with congenital myopathies and ERM or MH susceptibility.^[3,5,6]^ For the immunological studies, 20 MHS/ERM patients and 40 healthy controls will be included as previous studies in animal models showed an immunological gain-of-function in MHS mice in a lower number of subjects.^[12,13]^ The number of healthy controls is twice the number of MHS/ERM subjects as more variance is expected in the healthy controls than in the MHS/ERM subjects.

## 3 Discussion

Although MH and ERM are considered to be episodic manifestations of *RYR1* variants in otherwise healthy individuals, there is increasing evidence for neuromuscular and multisystem manifestation of disease in these patients. In the last decade, knowledge of the full spectrum of *RYR1*-related myopathies has significantly increased.^[2]^ However, the full disease spectrum and the effects of quality of life in affected patients are not fully understood yet. This study aims to explore in detail the degree and extent of neuromuscular and multisystem features of *RYR1*-related MH and ERM. Results of this study will improve recognition of multisystem symptoms in patients carrying *RYR1*-variants and contribute to optimization of the diagnostic work-up and counselling of patients with *RYR1* variants. This information will be of benefit to neurologists, anesthesiologists and geneticists working in the field of MH, ERM and *RYR1*. Furthermore, better understanding of the function of the RyR1 in the immune system and the immunological features of function in patients with *RYR1* variants will give more insights into immunological modifying factors relevant to the pathophysiology of ERM and MH.

A major strength of this study is the random selection of the study subjects from the MH and ERM cohort in two centers with large expertise regarding research and clinical care for patients with *RYR1* variants. Previous studies on this topic focused on special cases or a selected cohort which might have resulted in a significant publication bias. We will include subjects from the MH and ERM cohort including subjects who did not have a prior neuromuscular assessment before inclusion. Therefore, we will be able to include a representative study population for the MH and ERM cohort.

In conclusion, this study is expected to increase knowledge of the full spectrum of neuromuscular and multisystem features of *RYR1*-related MH and ERM and will establish a well-characterized baseline cohort for future studies on *RYR1*-related disorders. The results of this study are expected to improve recognition of *RYR1*-related symptoms, counselling and a more personalized approach to patients affected by these conditions. Furthermore, results will create new insights in the role of the immune system in the pathophysiology of MH and ERM.

## Acknowledgments

The authors thank the Canisius Wilhelmina–Radboudumc regional junior research funds for the financial support. Several authors of this publication are members of the Netherlands Neuromuscular Center (NL - NMD) and the European Reference Network for rare neuromuscular diseases (EURO-NMD).

## Author contributions

**Conceptualization:** Luuk R. van den Bersselaar, Marc MJ Snoeck, Nicol C. Voermans.

**Data curation:** Luuk R. van den Bersselaar.

**Formal analysis:** Luuk R. van den Bersselaar.

**Funding acquisition:** Marc MJ Snoeck, Nicol C. Voermans.

**Investigation:** Luuk R. van den Bersselaar.

**Methodology:** Luuk R. van den Bersselaar, Gert-Jan Scheffer, Lucas van Eijk, Ignacio Malagon, Stan Buckens, José AE Custers, Leonie Helder, Anna Greco, Leo AB Joosten, Nens van Alfen, Susan Treves, Marc MJ Snoeck, Nicol C. Voermans.

**Supervision:** Stan Buckens, Leo AB Joosten, Nens van Alfen, Marc MJ Snoeck, Nicol C. Voermans.

**Writing – original draft:** Luuk R. van den Bersselaar.

**Writing – review & editing:** All authors.
